# Modeling disease progression and treatment pathways for depression jointly using agent based modeling and system dynamics

**DOI:** 10.3389/fpubh.2022.1011104

**Published:** 2023-02-02

**Authors:** Syaribah N. Brice, Paul R. Harper, Daniel Gartner, Doris A. Behrens

**Affiliations:** ^1^School of Mathematics, Cardiff University, Cardiff, United Kingdom; ^2^Aneurin Bevan Continuous Improvement (ABCi), Aneurin Bevan University Health Board, Caerleon, United Kingdom; ^3^Department of Economy and Health, University of Continuing Education Krems, Krems an der Donau, Austria; ^4^Public Health Team, Aneurin Bevan University Health Board, Caerleon, United Kingdom

**Keywords:** system dynamics (SD) model, agent based modeling (ABM), depression-epidemiology, simulation modeling (SM), operations research

## Abstract

**Introduction:**

Depression is a common mental health condition that affects millions of people worldwide. Care pathways for depression are complex and the demand across different parts of the healthcare system is often uncertain and not entirely understood. Clinical progression with depression can be equally complex and relates to whether or not a patient is seeking care, the care pathway they are on, and the ability for timely access to healthcare services. Considering both pathways and progression for depression are however rarely studied together in the literature.

**Methods:**

This paper presents a hybrid simulation modeling framework that is uniquely able to capture both disease progression, using Agent Based Modeling, and related care pathways, using a System Dynamics. The two simulation paradigms within the framework are connected to run synchronously to investigate the impact of depression progression on healthcare services and, conversely, how any limitations in access to services may impact clinical progression. The use of the developed framework is illustrated by parametrising it with published clinical data and local service level data from Wales, UK.

**Results and discussion:**

The framework is able to quantify demand, service capacities and costs across all care pathways for a range of different scenarios. These include those for varying service coverage and provision, such as the cost-effectiveness of treating patients more quickly in community settings to reduce patient progression to more severe states of depression, and thus reducing the costs and utilization of more expensive specialist settings.

## 1. Introduction

Depression is a common mental health condition that affects approximately 4.4% of the world's population (1) with numbers dramatically on the rise since the onset of the COVID-19 pandemic. Depression cripples both the wellbeing of the individual and the society. At the individual level, this global burden reduces the quality of life (2), impairs physical functioning (3), and causes premature deaths (4, 5). At the societal level, depression damages the economy due to productivity loss (6, 7) and increased healthcare cost (8). Still, many countries fail to comprehensively understand their mental healthcare systems and the resources required to effectively operate these systems in the long run (9).

The literature has described a treatment pathway for various terms, such as care model, guideline, care map, multidisciplinary care, integrated care pathway, protocol, evidence-based care (10), and possibly many more. It encompasses four criteria that the intervention *1. is a structured multidisciplinary plan of care; 2. is used to translate guidelines or evidence into local structures; 3. details the steps in a course of treatment or care in a plan, pathway, algorithm, guideline, protocol or other ‘inventory of actions' (i.e., the intervention exhibits time-frames or criteria-based progression); 4. aims to standardize care for a specific population* (11) such as those living with depression.

Standardized treatment pathways guide the best practice regarding therapeutic interventions for persons with depression, for example, National Collaborating (12). The cross-relationship between pathway design and the individual's disease progression along this care pathway (especially when the system is overwhelmed) is not explicitly considered. Shedding light on this issue and its implications for healthcare management and public health are the focus of this article.

The studies on treatment pathways have used various methods, including computer simulation: discrete event simulation (DES), system dynamics (SD), and agent-based modeling (ABM). DES is a simulation tool that models queueing systems. A system is represented by a network of queues and activities. Entities move from one activity to another in discrete time steps (13). SD is regarded as a continuous simulation method that originated in system engineering introduced by see, Forrester (14). SD models a system as a network of its subsystems where entities with similar characteristics flow continuously. Whereas, ABM is a simulation approach that model individual agents that make up a system. These agents are unique, can make decision, and can interact with other agents or the environment they live in Railsback and Grimm (15). The application of DES, SD, and ABM in healthcare in general and in mental healthcare is described in subsequent sections on the related work.

Although these simulation methods have been used in healthcare modeling, it is still small in number where studies have used a combined method. We, therefore, introduce a hybrid approach marrying agent-based modeling (ABM) and system dynamics (SD). In this context, *hybrid* means combining two or more simulation methods (16) in a single study where advantages from both methods enrich the overall model. Hybrid simulation has been implemented in studying healthcare systems and found that it offers flexibility and efficiency in also capturing behavioral elements in the system (17). The basic concepts of SD and ABM methods are introduced in Section 3 where we describe the model building.

The multi-agent part of our model mimics individual patients moving through disease stages in response to (personal choice and) timely availability of appropriate treatment. The SD part of the model represents the complex treatment pathways associated with each severity level of depression. We integrate both models by constructing several linking points. The number of individuals having depression will impact healthcare capacity. Conversely, the limitation of sufficient healthcare resources will ultimately alter the progression of depression in individuals. Our approach helps us understand how to holistically design and manage service provision with the patient at the system's center, which has been unexplored in the context of depression so far. The novelty, in modeling terms, is to explicitly integrate links between the structure of the care system and the individuals' pace of transitioning through it. Also, we can learn [on the strategic level, for a taxonomy, see Hulshof et al. (18)] the capacity needed in the system that prevents harming patients by delaying care. This, in turn, enables the assessment of the future expected burden for the healthcare system when resources are (temporarily) insufficient to meet service demand. Finally, (on the operational level), our model helps understand patients' pathways to better manage queues enabling rapid access to services.

Our computational results, informed by data from the UK's National Health Service (NHS), indicate that patients accessed general practitioners (GPs) more often than specialist services. The increase in service coverage did reduce not only depression progression but also reduced the use of inpatient services. The results support the recommendation to provide mental health services in the community, which ultimately reduces the use of specialist settings.

## 2. Related work

The literature studies have investigated the application of discrete event simulation (DES), system dynamics (SD), and agent-based modeling (ABM), in healthcare settings [see Fone et al. (19), Jun et al. (20), Günal and Pidd (21), Gönül-Sezer and Ocak (22), Brailsford et al. (23), and Katsaliaki and Mustafee (24)]. Various single methods, including simulation, have been applied in mental health-related studies (25).

With respect to mental health, DES has been applied in studies related to cost-benefit analysis for comparing different treatments or drugs (26–29); modeling disease course of schizophrenia (30); the efficacy of a therapy (31); patients flow capturing links between different healthcare services (32–36); staff configuration; and best service hours (37). It appears that either in the area of healthcare system operation or healthcare system design and planning, patient flow modeling using DES has been shown to be a significant aid for the decision-makers to find the best possible service structure. The problems related to the process flow being modeled shared a similarity, that is, patient's waiting time in conjunction with limited available resources. DES has been implemented to inform evidence-based structural change in providing an efficient mental healthcare service.

### 2.1. System dynamics modeling in healthcare and mental healthcare

System Dynamics (SD) has been regarded as a modeling technique for a complex system (38). A complex system is described using feedback loops and delays, and the structure of the interconnected elements in the system will determine the behavior of the system (39). SD can accommodate overlapping boundaries of a system which allows incorporating all dynamics necessary to be modeled (40), such as found in healthcare system (41). Furthermore, SD does not require a large and detailed data. Individuals in the population being modeled can be aggregated which makes SD suitable for problems at the strategic level (41, 42).

The applications of SD in general healthcare have addressed problems related to patient flow in an emergency and urgent care (43–45); patient flow through different services from ED to hospital wards (46) or through wider healthcare and other care services such as acute hospital, home and community care, rehabilitation and complex continuing care, and long-term care (47).

In mental healthcare, studies have addressed problems such as implementation of a treatment strategy in a mental health facility (48, 49); cost-benefit analysis at the same time capturing different sectors (treatment and recovery, therapist, and labor market) (50); patient flow from GP to hospital (46); mental health in prison and forensic sectors (51), in military system (52), or community center (53); disease progression by incorporating factors associated to depression (54) or by describing different parts of brain in schizophrenia (55).

### 2.2. Agent-based modeling for disease progression

Agent-based modeling (ABM) focuses on representing agents with their individual behaviors and connections with other agents and their joint environment (15, 56). ABM capable of capturing the complex characteristics and behavior of the systems and addresses problems relating to systems' emergent behavior (57). For example, if the agent represents patients, their characteristics such as sex or different age groups can be incorporated. Moreover, patients' decision such as adhering certain medication regime or those resulting from interaction with other agents in the model (such as doctors, nurses, or other patients) can also be modeled. The developed model may give new insights resulting from the decision made by the patients, though a useful insight is not a requirement to build an ABM (58).

The applications of ABM in healthcare setting have addressed complex issues relating to service quality, economics, and workloads (59); process flow in reducing patients' waiting time (60); and disease spread such as cholera in a refuge camp (61). Agents such as patients and healthcare professionals can have static interaction, where their behaviors are not affected by the environment (62), or a dynamic interaction where their decisions are based on the joint environment (59).

In mental healthcare-related studies, ABM can capture three different levels of agents (individuals, organizations, and society) (63). This type of modeling approach incorporates the population mental health status, while at the same time assuring the quality of care service. The characteristics of individuals may relate to factors such as sociodemographics, the neighborhoods where the individuals reside and the traumatic life experience (64). The complex care system being modeled can represent different settings such as social and criminal justice (65).

Area of applications in mental health include optimal allocation of resources in relation to prevention strategy with certain treatment in mental health (64); development of disease in relation to stigma and social exclusion (66); and comparing different treatment models (67).

### 2.3. Hybrid simulation in healthcare

Frameworks for developing a hybrid simulation model has been proposed in the literature. Combining SD and DES, can be done in three possible ways: hierarchical, process-environment, and integrated (68). Studies have used a combination of simulation techniques (ABM, SD, and DES) in modeling healthcare systems. Complex dynamics of healthcare system can be modeled in a more efficient way using hybrid simulation (17). Hybrid simulation studies are still few in numbers and mainly used to address problems related to system operational and strategy (69).

The hybrid simulation has been applied in areas such as clinical medicine (70); spread of disease in conjunction with different interventions (71); healthcare technology assessment (72); and forecasting population growth for healthcare demand (73). Hybrid simulation studies have used SD for modeling population dynamic in relation to disease progression, DES for representing healthcare clinics, and ABM was used for modeling individuals with characteristics such as age, gender, and behavior (e.g., interaction between individuals).

In conclusion, our review of related work highlighted studies that modeled mental health illness and its care using different methods. Some focused on the depression progression and others focused on modeling the system of care. The literature suggested a gap in using a combined method to study problems related to mental health and the care pathways.

## 3. A hybrid model of depression

### 3.1. The system dynamics model for treatment pathways

The System Dynamics method has two essential building blocks being stocks and flows, and feedback. Stocks represent the accumulations and are influenced by the inflows and outflows. Diagrammatically, stocks are represented by rectangles, whereas flows are represented by pipes with arrows pointing toward the rectangles (inflows) and out of the rectangles (outflows). Flows have valves that control them (i.e., control the amount of entities/materials/things go into and out of the stocks). The pipes, representing the flows, attached to clouds represent the sources and sinks for the stocks. Behind the valves are where we formulate the equations governing the behavior of the stocks. The net rate of change in any stock is the difference between the inflows and the outflows. Mathematically it can be defined using differential equation as:


$$\frac{d\left(Stock\right)}{dt}=Inflow\left(t\right)-Outflow\left(t\right)$$

Different feedback structures may arise from the complexity of the structure of the stocks and flows. For example, a simple structure may give positive feedback which can generate exponential growth or decline, whereas a more complex structure can give rise to oscillation. A more detailed reading on SD modeling can be found in (39).

#### 3.1.1. Model conceptualization

The purpose of a SD model is to represent the treatment pathways for depression. Our model can be used to capture information on individual flows through the system. SD is considered in this study due to its advantages in dealing with complex systems and when detailed data are not available (74). Moreover, the treatment pathways represent the situation when an individual agent in and agent-based (AB) model is in the treatment state. An individual can use different services to receive treatment. It would be challenging to further add in treatment pathways to the AB model that itself is already complex.

The treatment pathways for depression is divided into three categories based on severity levels (mild, moderate, and severe). This division is necessary due to several reasons. First, different levels of severity may need different management of depression. Second, developing dedicated pathways for each level of conditions assists in obtaining analysis results according to the specific condition. Third, it provides a clear description of the interaction points between the disease progression model (in ABM) and the system of care model (in SD) for each severity case.

#### 3.1.2. Model assumptions

Modeling the system of care for depression, in this study, rests upon recommended WHO TOLKIEN II pathways (75). These pathways were developed based on the collected data of people suffering from mental health conditions, evidence on the best treatment for each condition, and extensive expert opinions on the best treatment structure (75). WHO TOLKIEN II pathways provide a clear description of depression treatment for each severity level (mild, moderate, and severe depression), which is based on a stepped care model and in line with NICE guidelines (12). It follows, the description of the treatment pathways refers to the two resources (12, 75).

We model treatment for mild depression to be managed in primary care. A person's GP, as the first point of contact, will provide four consultations along with other resources to help patient manage their condition. The majority of those who develop mild depression symptoms will remit within 6 weeks. Those patients whose condition persists beyond this time period are offered to choose between taking medication (under the supervision of their GP) or receiving therapy managed by a clinical psychologist. It is perceived to start with generic medication (NICE recommendation) with a duration of 46 weeks (TOLKIEN II). Patients are allocated up to six GP visits during the medication treatment. For those patients who receive therapy, they are allocated up to six clinical psychologist visits. After having received clinical psychologist treatment, patients check in with their GPs to ensure that the GPs keep track of the patient's progress.

Up to this point, the care pathways are similar for mild and moderate depression. Some additional treatment for people with moderate depression after this point is made to either continue the treatment with a clinical psychologist for more intensive therapy or to visit a psychologist for more intensive treatment with medication. At the end of the treatment, people will visit their GP for a final evaluation.

**Figure 1:**
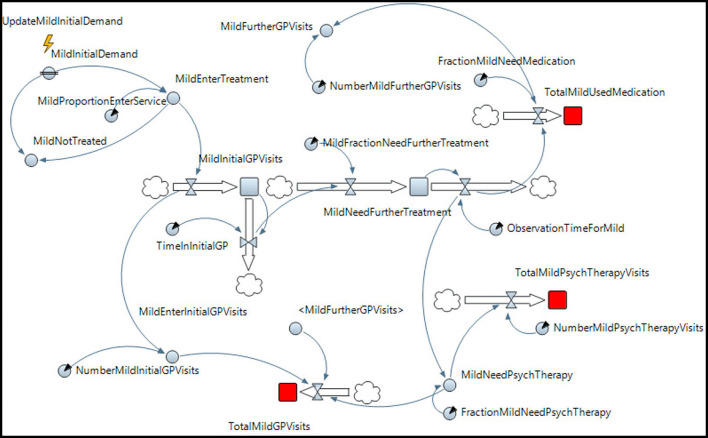
SD model for mild depression.

Mild and moderate depression, according to this treatment recommendation, will not need any inpatient care or management from a mental health team. Their care is managed entirely through outpatient services. Figure 1 describes the corresponding SD model built in AnyLogic. (Note that the models for moderate and severe depression are provided in the Supplementary material).

Recommendations differ for individuals with severe depression. Their treatment pathways are more complex, involving inpatient care as well as a mental health team. People with severe depression share similar care pathways to those with moderate depression for the majority of their treatment. However, in the case where inpatient care is needed due to high risk of suicide or difficulty in managing the patient in more open facilities, inpatient care and mental health team services are then needed.

The treatment pathway for severe depression also admits the possibility that some people might need to be admitted to the inpatient facility at the initial stage. This creates additional need for inpatient facilities.

In practice, the community mental health teams (CMHTs) work in partnership with other health services including GP and inpatient care. CMHTs receive referral from the GP, acute inpatient mental health team, and other mental health teams. They also make referral to other health services such as the inpatient care in the emergency cases. Together with the inpatient care team, CMHTs work to provide the right level of care and support for the individual upon discharged from the hospital. CMHTs provide service to individual with mental health condition at the clinic or at patient's home. The service provided by the CMHTs outside the inpatient care help minimize the length of stay in the inpatient care. Our model capture the services provided by CMHTs for those individuals with severe cases at two points: at the start of the treatment and when an individual is admitted to the hospital as emergency cases.

Our assumption is that the developed model was intended for adult patients only, which tallies with the recommendation proposed by the NICE. In system dynamics modeling, entities flow in the model are homogeneous. In our model, the "adult" patients formed a homogeneous entity moving from one health service to another in the treatment pathways. Their differences, in severity level in this case, were represented by different treatment pathways for each condition.

#### 3.1.3. Model parameters

The parameters used for the SD model mainly come from the recommendation pathways (12, 75). Supplementary Table S9 summarizes the parameters and their sources.

### 3.2. Using ABM to map individual disease progression

**Figure 2:**
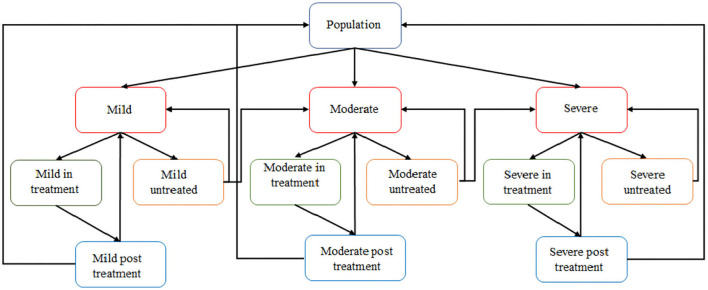
Illustration of multi-agent depression model built in AnyLogic.

The entities of our multi-agent simulation model represent people with specific characteristics, aged 18 and above. Each individual (or agent) can be in one of the four health-related states at a time: (i) no, (ii) mild, (iii) moderate, or (iv) severe depression (see Figure 2). At each disease stage (mild, moderate, and severe), individuals are then in one of the three care-related states: (i) untreated, (ii) in treatment, or (iii) discharged after treatment.

We omit direct interaction between individuals (as depression is not an infectious disease) but integrate interaction of the individual with the healthcare system. Through this interaction, we will capture the effect of seeking and receiving treatment on disease progression. The latter is how individuals affect each other indirectly and explains how the thresholds induced by collectively using the system feed back on individual pathways of disease progression.

At the starting point *t* = 0 of the simulation, none of the *n*_0_∈ℕ individuals in the population suffers from depression (or is not yet diagnosed). At each time step *t* (*t* = 1, ..., *T*), individuals can develop the disease, where time progresses in weekly steps. The transition to (1) mild, (2) moderate, and (3) severe depression is governed by the estimated prevalence of depression in the population (represented by *p*∈[0, 1]) and the estimated proportions in developing respective symptoms, labeled as *a*_1_, *a*_2_, *a*_3_∈[0, 1]. Those suffering from depression at time step *t*, generate demand for the healthcare service, which will be explained by means of the SD model to be presented later.

Once having developed depression, the transition probabilities to deteriorate (*s*^*ij*^∈[0, 1]), to become disease-free (*r*^*ij*^∈[0, 1]), to get worse more than 6 months after ending treatment (*u*^*ij*^∈[0, 1]), or to die (*d*^*ij*^∈[0, 1]), determine an individual's modeled future (with *i, j*:*i*∈{1 = *mild*, 2 = *moderate*, 3 = *severe*} and *j*∈{1 = *untreated*, 2 = *discharged*}). Note that in practice, *r*^*i*1^ is usually referred to as *remission* probability, and *r*^*i*2^ as probability of *recovery*. Moreover, *u*^*i*1^ is often called *recurrence* probability, while *u*^*i*2^ is denoted as *relapse* probability. *s*^*i*2^ is not considered in the model.

The number of individuals who suffer from depression and can access healthcare services during week *t* (denoted as $n_{t}^{i2}\in \mathbb{N}$) will change their state to “in treatment.” They will remain in this state until treatment is finished, which will change their state to “discharged.” Their number is represented by $n_{t}^{i3}\in \mathbb{N}$. People experiencing symptoms of depression without receiving treatment during week *t* will be labeled as untreated (represented by $n_{t}^{i1}\in \mathbb{N}$). The underlying rates for being “in treatment” or being “untreated” necessary to compute $n_{t}^{ij}$ are taken from the results generated by the SD model to be described later.

The parameterization of the AB model is summarized in Table 1. Recall that transition rates are scaled to time steps of 1 week.

**Table 1:** Parameter values for multi-agent simulation model of disease progression.

**Parameter**	**Value**
Initial population size	*n*_0_ = 5,000
Time horizon	*T* = 2 years
Prevalence of depression	*p* = 7.7%^a^
Proportions of severity levels for the disease	*a*_1_ = 29.4%, *a*_2_ = 38.8%, *a*_3_ = 31.8%^b^
Probability of deteriorating from mild to moderate depression	*s*^11^ = 7.0%^b^
Probability of deterioration from moderate to severe depression	*s*^21^ = 6.0%^b^
Recovery (treated mild, moderate, severe)	79.3%, 64.5%, 54.9%^b^
Recovery (untreated mild, moderate, severe)	81.7%, 74.7%, 57.8%^b^
Recurrent (untreated, treated)	33%, 14%^c^
Death rate per 100,000 population, for all conditions	*d*^*ij*^ = 1, 045.7 for *i* = 1, 2, 3;*j* = 1, 2^d^
Treatment duration in weeks for mild depression	Triangular (4, 26, 52)^e^
Treatment duration in weeks for moderate depression	Triangular (4, 26, 56)^e^
Treatment duration in weeks for severe depression	Triangular (26, 52, 76)^e^

^*a*^Est. using depression registers and mid-population estimates for adults (76, 77).

^*b*^Adapted from Table 2 in Simon et al. (78).

^c^Source (79).

^*d*^Source (80).

^*e*^Source (12, 75). The mean of 26 weeks is taken from Üstün et al. (81) and Posternak and Mille (82). For severe, 52 weeks is the duration of treatment when the possibility of 30% of people appear to respond to any treatment in Andrews and the TOLKIEN II Team (75).

### 3.3. Combining AB and SD models

The developed AB and SD models are connected so that they can run synchronously. The purpose is to investigate the effect of depression prevalence to the healthcare services and the provision of care to the progression of depression. To realize this purpose, the combined model has three connection points for each severity level and one additional point for severe depression treatment pathways.

The AB model will generate estimation on the number of people falling into one of the depression conditions (mild, moderate, and severe). The assumption is that when individuals are in one of the three depression conditions, the conditions are known to them but they have not made any contact to the care services. The number of individuals generated from AB model will be forwarded to the SD model.

The SD model has limited service coverage, hence, not all individuals with depression conditions will enter the treatment pathways. The number of individuals entering and not entering the treatment will be sent to the AB model. These two numbers will be used to move individuals to being in treatment or not treated accordingly. The design of the connection between the AB and SD models has been taken into consideration the different modeling paradigms of the two techniques. In the context of the study, the information on the number of people entering or not entering the treatment pathways is used to choose the exact number of individuals in AB moving to being in treatment or not treated.

**Figure 3:**
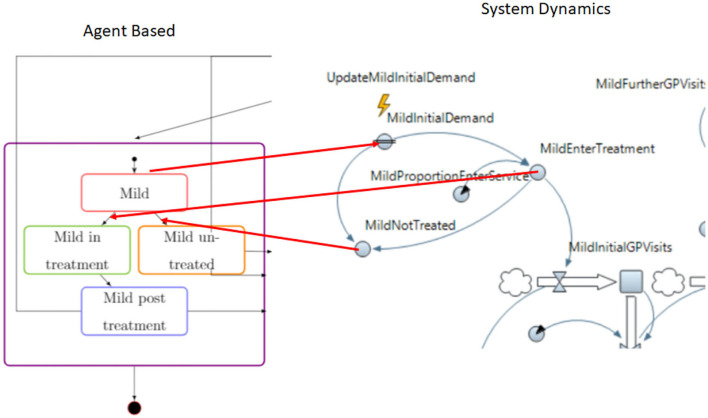
Combining AB and SD models.

Figure 3 illustrates the connection between the AB and SD models.

### 3.4. Model implementation

The integrated depression model outlined earlier was developed in AnyLogic 8 University (version 8.3.3 Copyright(c) AnyLogic, North America). AnyLogic is the leading software which is capable of combining two or three different simulation methods (ABM, SD, and DES) in one platform. The population size is *n* = 5000. The time unit and all necessary parameters were scaled to week. The simulation was run for 104 weeks with warm-up period of 26 weeks. The run time was set to 50 and the replication per iteration is 10.

The experimentation has three scenarios representing different service coverage (47, 65, and 80%) which were adapted from the statement by NICE (83). The service coverage of 47% was set as the baseline scenario.

The model was run with different service coverage for four main purposes. The first purpose is to get the prevalence of depression at different severity levels. For this purpose, the number of people with depression was recorded for each depression type.

The second purpose is to find out the effect of the different service coverage on the progression of depression. The model captures two different types of disease progression namely the progression from mild to moderate depression and the progression from moderate to severe depression. The expectation is that the model will show the higher the service coverage, the slower the progression of depression.

The third purpose is to monitor the effect of different service coverage on the prevalence of relapse cases in each of the severity levels. The model captures the relapse cases from both conditions (i.e., from being treated and not treated). For this purpose, the expectation is that the model will show the more people with depression that get treated, the less the occurrence of relapse cases.

The fourth purpose is to estimate the burden of depression. This purpose is achieved by evaluating the costs related to the healthcare use and the disability adjusted life years (DALYs).

### 3.5. Structural validation

The testing procedures involve checking the input–output from the model results correspond to the parameters used to run the model (84), ensuring that the developed model is representative of the conceptual model (13, 16). The testing was conducted at each individual sub-model (AB and SD) as well as overall model to gain confidence on the model (85).

The conceptual model and the initial results from the model were presented to the experts in Aneurin Bevan University Health Board (ABUHB). The critical review was sought on the model structure which then improved the model to be more representative. This includes the additional service type, crisis resolution home treatment team (CRHTT), which was not represented in the initial model. Another point highlighted from the review was to add the link between the mental health team and the psychological therapy and psychologist, which also represents the flow of patients accordingly.

The aim for testing the AB model is to compare the prevalence rate generated by the model with the prevalence rate used from the population. The overall depression prevalence rate used in the model is 7.7% and the proportion of mild, moderate, and severe depression is 29.36, 38.79, and 31.85%, respectively. The calibration process was also conducted to adjust the rate used in running the model. The comparison tests for the proportion rates for all the severity levels did not show significance.

The testing for the SD model was conducted by checking the accumulation points where the patient receives treatment. The setting of the service coverage was set for 0 and 100%.

The testing for the hybrid model aims to investigate whether the connection between AB and SD models works as intended. The prevalence of depression (for each category of severity) in the AB model is used to generate demand for the SD model. This demand is updated each week and sequentially the SD model computes the rate of people entering the service. Since the rate is defined to be the key element in determining the size of demand entering the service, this process yields two rates; one for entering the system of care and the other for not entering the system of care. The number of people enter and not enter the treatment in the AB model was compared with the number generated in the SD model.

## 4. Results

### 4.1. Estimating the population with depression

Table 2 presents the model output for the average yearly mean prevalence for mild, moderate, and severe depression. Levene's test for equal variance and the Shapiro–Wilk for the normality test indicated insignificance. Based on this, the ANOVA test was conducted, and the results showed that there is not enough evidence to suggest that the mean prevalence for each severity are different by different service coverage. This suggests the stability of the model in generating the prevalence for depression.

**Table 2:** Average depression prevalence by severity and service coverage.

**Service**	**Mild**	**Moderate**	**Severe**
**Coverage**	**Mean (95% CI)**	**Mean (95% CI)**	**Mean (95% CI)**
45%	112.34 (111.65, 113.02)	148.36 (147.60, 149.12)	121.03 (120.38, 121.67)
65%	112.34 (111.68, 113.00)	147.85 (147.12, 148.58)	121.41 (120.75, 122.08)
80%	112.36 (111.74, 112.98)	148.75 (147.98, 149.53)	122.24 (121.57, 122.91)

Scaling the results to population in each health board in Wales yielded a 1 year estimate for the number of people with depression. It follows that the higher the population size in a health board, the higher the estimate is. Table 3 presents the estimation from scaling the simulation results.

**Table 3:** Depression prevalence projected to population in Wales by LHB.

**Context**	**Population size**	**Min**	**Median**	**Max**
Model	5,000	304	393	500
Aneurin Bevan	476,139	28,902	37,377	47,614
Betsi Cadwaladr	571,244	34,675	44,843	57,124
Powys	111,070	6,742	8,719	11,107
Hywel Dda	318,593	19,339	25,010	31,859
Abertawe Bro Morgannwg	437,054	26,529	34,309	43,705
Cwm Taf	242,199	14,701	19,013	24,220
Cardiff and Vale	399,772	24,266	31,382	39,977

**Figure 4:**
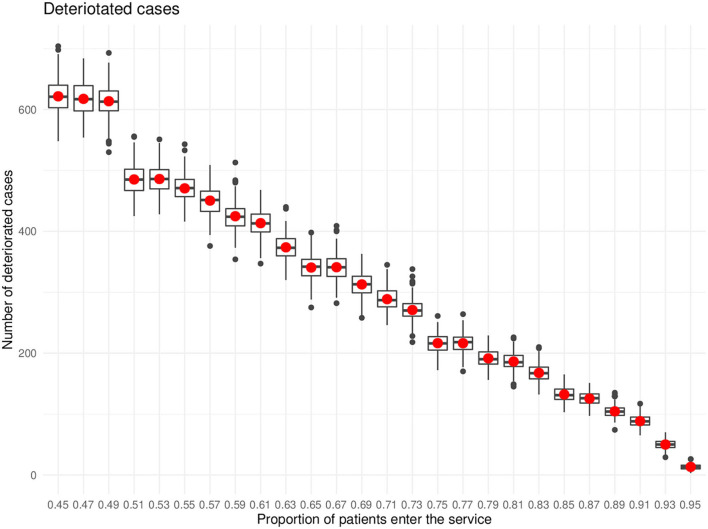
Deteriorated cases.

The progression of depression was recorded in two types: from mild to moderate and from moderate to severe. The results showed that the higher the service coverage the lower the number of people with depression progressed from lower severity to higher severity levels. The Kruskal-Wallis test followed by a *post-hoc* test at 99% confidence level showed significance. Figure 4 illustrated the deterioration cases against different percentages of service coverage.

**Figure 5:**
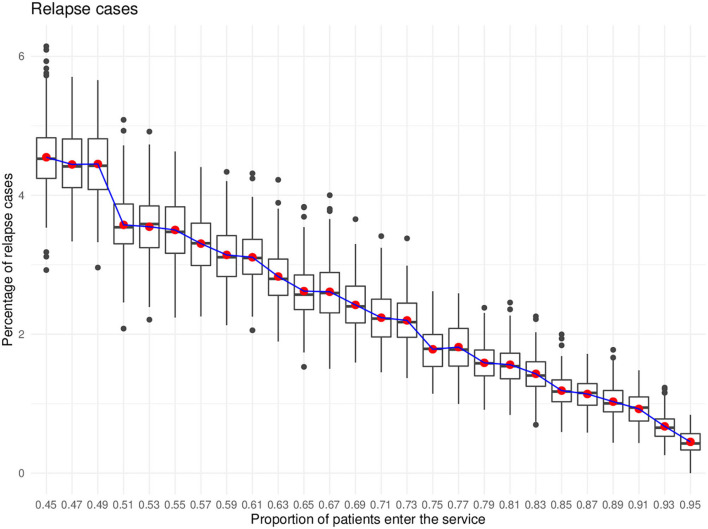
Relapse cases.

The effect of different service coverage on the number of people with relapse cases was investigated. The statistical tests suggested that the number of relapse cases was reduced, in the case of mild and moderate depression, with higher service coverage. In the severe depression cases, the number of relapse cases was not affected by different service coverage. Figure 5 illustrated the percentage of relapse cases against different percentages of service coverage.

The model was used to estimate the service needs. By the service, it means that the components incorporated into the model include GP, psychological therapy, medication, psychiatrist, community mental health team, crisis resolution home treatment team, and inpatients facilities. Each component of the model has a unit measure corresponding to it. Table 4 illustrates the typical 1 year service needs from the simulation run.

**Table 4:** Typical simulation results for mental health service use with different service coverage.

**Service**	**Average**	**GP**	**Psych. Therapy**	**Medication**
**Coverage**	**prevalence**	**(visits)**	**(visits)**	**(people)**
	**Mean (95%CI)**	**Mean (95%CI)**	**Mean (95%CI)**	**Mean (95%CI)**
47%	381.72 (380.51, 382.93)	1031.11 (1026.02, 1036.20)	180.09 (179.28, 180.97)	58.45 (58.16, 58.74)
65%	381.60 (380.44, 382.75)	1746.90 (1740.51, 1753.29)	301.80 (300.73, 302.87)	99.21 (98.84, 99.58)
80%	383.35 (382.22, 384.48)	2105.07 (2097.72, 2112.41)	363.55 (362.32, 364.77)	119.58 (119.16, 120.00)
**Service**	**Psychiatrist**	**CMHTeam**	**CRHTT**	**Inpatient**
**Coverage**	**(visits)**	**(visits)**	**(people)**	**(weeks)**
	**Mean (95%CI)**	**Mean (95%CI)**	**Mean (95%CI)**	**Mean (95%CI)**
47%	126.97 (126.02, 127.91)	387.06 (384.27, 389.85)	82.63 (82.18, 83.09)	75.90 (76.42, 77.38)
65%	216.23 (215.06, 217.40)	615.66 (612.12, 619.20)	92.94 (92.44, 93.43)	58.61 (58.15, 59.08)
80%	260.04 (258.62, 261.45)	722.81 (718.60, 727.02)	93.54 (92.99, 94.10)	43.05 (42.58, 43.51)

The results from parametric tests indicated significant difference in median across the different service coverage in each service type. Further analysis revealed that all but a comparison between 65 and 80% on the community resolution home treatment team showed significant. This implies that the different service coverage may lead to the different volumes in service needs. The trend shown in Table 4 indicates that the increase in the service coverage will increase the service use, except for the use of the inpatient facility.

The results from the service needs can be used to investigate the implication of different service coverage to the healthcare costs as well as to DALYs measures. To evaluate the impact of different service coverage, the study used healthcare costs estimated by Curtis and Burns in Curtis et al. (86). The unit measures and the results are presented in Supplementary Table S2. The results highlighted that the increase in the service coverage increases the healthcare costs in many areas except the costs of using the inpatient facilities. The apparent results can be seen between the 65 and 80% service coverage.

Disability adjusted life years (87) can be estimated using disability weight of 0.145, 0.396, and 0.658 for mild, moderate, and severe depression, respectively. When comparing DALYs averted between two different service coverages, it indicated that the biggest DALYs averted is gained from increasing the service coverage from 47 to 80%. Whereas, the smallest DALYs averted is from the difference between 65 and 80%. The findings, from estimating DALYs averted and potential reduction in the inpatient care costs, imply that increasing the coverage up to 80% is better than 65% in this case, should such increase is possible.

## 5. Discussion and conclusion

### 5.1. How can we build a hybrid simulation model which addresses depression progression and its related treatment pathways?

The study combined two methods: the agent-based modeling was used to model depression progression, and the system dynamics was used for describing the treatment pathways related to depression. The two models were connected to run synchronously to investigate the impact of depression progression on the health services and how the limitation in the services may impact the progression of depression.

The depression progression was modeled using different health categories mainly mild, moderate, and severe. These categories have been used in studies to model the population affected by depression (65). Studies varied in differentiating the condition. Some are more elaborate by including relapse, response to treatment or recovery and death (26). The present study includes categories that have been found in the literature and elaborated the states to include being in treatment, untreated, and out of care. These deemed necessary to capture the effect of receiving treatment to the progression of depression. In modeling a disease progression where categorization is not available, but individual patients' data are sufficient, a machine learning method can be used to classify the patient's condition [see an example in Llamocca et al. (88)]. The categories found in the data then can be used to inform the AB model.

The use of the agent-based model to generate demand for the healthcare services is similar to what has been done in studies using different methods such as Markov models. The characteristics of the agent-based methodology suit its use in this regard. It offers flexibility in modeling a patient with health condition, the complexity of which can be added, providing the data is available, without having to change the structure of the model (74).

The treatment pathways were modeled using a system dynamics and were based on the recommended stepped-care treatment pathways (12, 75). The literature found studies that modeled treatment pathways in a healthcare clinic using the stepped-care (49) or that has captured several different services (46).

The flexibility of SD in capturing complex network of healthcare is suitable for the current study. The care services, where patient received the treatment, were described as stocks in the model. The developed SD model captured the flow of patients and quantification at any service can be done by measuring the accumulation of number of patients using the service.

The use of SD to model the treatment pathways, though relatively simple in concept, complemented the AB model. The treatment pathways turned out to be complex and such complexity would not be easily added to the AB model. The design used in this study, where individuals are modeled using ABM and the health system is modeled using SD, has been implemented elsewhere (89). Such design may be referred as “process-environment” (68) which has been used in the studies related to healthcare (16).

The study captured healthcare services from GP to hospital inpatient. The inclusion of mental health team and other specialists add toward the comprehensiveness of the complex healthcare service modeling. Though this may be ideal to model, it was difficult to implement and only went as far as a model conceptualisation of a system of care. Furthermore, the challenge that arises from modeling a complex healthcare system is finding the parameters to run the model.

### 5.2. Using a recommended treatment model, how can the prevalence of depression affect healthcare services?

The developed model captures the flow of patients who received treatment. The treatment recommendation suggests to start the treatment with lower intensity, and this is administered in a GP. The simulation results highlighted that GPs were accessed more than specialist services, which confirms what has been found in the literature (90). Indeed, in many countries, GPs serve as gate keepers to a more specialist service in healthcare system.

The results highlighted that the increase in service coverage has increased the service use (including the use of medication) which ultimately increase the healthcare costs. This finding may not be surprising in practice, because as more people access the health service, there will be an increase in the use of the service. However, the results also suggested that the increased service coverage reduced the inpatient care use. This supports the recommendation to treat mental health patients in a community to reduce the utilization in the specialist settings (91).

The developed model captures different mental health services from community mental health team to hospital inpatient care. The collaboration care in mental health services is essential to better managing the chronic depression condition which can be treatment resistant (92). It is found that collaborative care management in primary care can reduce the time to remit for depression (93).

### 5.3. How can different levels of service coverage affect the progression of depression?

The results indicated that the progression of depression from moderate to severe showed a better response compared to the progression from mild to moderate with the increase of service coverage. However, in general, the results indicated that the higher the service coverage the less the progression of depression to more severe conditions.

Another highlight from the results showed that people with mild and moderate depression responded better with the increase of service coverage compared to those with severe conditions. This may suggest that increasing the service coverage may help in preventing people with mild and moderate depression to relapse. Studies have found that receiving treatment, medicine, or therapy, whether short or long term, prevents relapse (94–96).

On the other hand, the relapse cases in severe depression are found to have no effect with different service coverage compared to mild and moderate depression. This might be explained by the fact that severe depression is a chronic condition which can be treatment resistant (97, 98), and relapse can still be experienced even after remission from receiving treatment (99). Anti-depressant medication alone, though being the common choice for depression, might not be sufficient to treat those with treatment resistant depression and might have to be combined with other more effective therapies such as electroconvulsive therapy (ECT) (100).

### 5.4. Reflection on the model, limitations, and recommendations for future study

The mental health policy and strategy such as provided in the context of Wales (101–103) all focus on delivering mental health services which take into account individual patient's needs based on the demographic profile, social, cultural, and financial. Patient's decision-making on choosing a treatment should be incorporated into providing a treatment that aims to alleviate the burden and prevent the condition from developing into the worst condition. While at the same time, the system of care should be monitored continuously its effectiveness and efficiency.

The literature showed studies that have used simulation methods to study mental healthcare-related problems. The current study showed the possibility of the hybrid simulation to be used for modeling a mental healthcare which take into account the patient and the system perspective. The model can provide supports to the points highlighted in mental health strategy and policy in evaluating a mental healthcare. The model can be used to explore the impact of changing a certain policy to other elements in the system.

The study demonstrated the implication of changing the service coverage in mental healthcare to the progression of depression and vice versa. However, developing such complex model faced challenges. First, the treatment pathways vary from context to context. The current study used treatment pathways provided by a national guideline. Although the guidelines were developed based on the extensive and rigorous methodology, the implementation itself is questionable. This highlights the need of standardized treatment pathways specific to the context.

Second, the parameterisation of the model came from different sources. It is not possible to acquire detailed data, which can provide information on disease progression as well as the use of healthcare, from one context. This is due to the fact that data collection in healthcare is fragmented. Although in some context it is possible to link data from different sources, the accessibility of such data may be difficult. This highlights the need of data collection that captures the progression of disease and well as the use of healthcare service. Detailed data on patients with mental health will allow to model the patients with their behavior as well as their individual disease progression. If the agent-based model is used, the connection between patients and health professionals, as well as other cares, can be incorporated.

Third, the system model was developed based on the treatment pathways which mainly include the clinical settings. Studies have found that people suffering from mental health conditions may affect other services, such as social support, housing support, and the justice system. For example, a study by Smith et al. (51) includes mental health in prison and forensic sectors. The challenges faced in the current study in developing a complex healthcare system may explain why there are limited number of studies on complex healthcare systems that have been quantified and calibrated using the real data.

Despite challenges faced in developing a complex hybrid model, the study sheds some insights. It is feasible to combine different methods in one model that capture individual patient condition as well as related treatment pathways. This hybrid method can offer great potential in evaluating health service performances as well as monitoring the impact of service provision to the patient's health condition. Since the model was built based on the national recommendation and parameterized using a context within the UK, it is possible to repeat the study in other areas of the UK providing sufficient data are available. In general, the model can be implemented in different contexts from different countries where similar treatment pathways are implemented. Separating the patient's condition from the treatment pathways provides a convenient way to modify the model. It is possible to amend the treatment pathways that suit the context by modifying the SD part, without having to modify the AB part.

### 5.5. Conclusion

The literature and the current study have shown that the combination of two simulation methods to address healthcare problems offers an interesting possibility. In particular, when the problem being addressed incorporates the disease and treatment pathways. The challenges, however, were found which relate to both the structure and the parameterization of the model. This leads to a question, can a sophisticated hybrid simulation ever be built to describe a complex healthcare system? It seems that the genuine engagement of the experts from academic and healthcare disciplines and the availability of data from a single context being evaluated are the key ingredients.

## Data availability statement

The original contributions presented in the study are included in the article/Supplementary material, further inquiries can be directed to the corresponding author.

## Author contributions

SB carried out the modeling, data analysis, gathering of experimental results, and drafted the manuscript. DB, DG, and PH were overseeing the modeling, data analysis and interpretation of results, and worked on manuscript improvements. All authors contributed to the article and approved the submitted version.
