# Supplementary material for: Modeling disease progression and treatment pathways for depression jointly using agent based modeling and system dynamics
Source: Front Public Health. 2023 Feb 2;10:1011104. doi: 10.3389/fpubh.2022.1011104 (PMC9932262; doi:10.3389/fpubh.2022.1011104)
Supplement: Supplementary file 1 [file Data_Sheet_1.pdf]

Supplementary Material

1 SUPPLEMENTARY TABLES AND FIGURES

Table S1. Typical simulation results for mental health service use with different service coverage.

Service Coverage	Average prevalence Mean (95%CI)	GP (visits) Mean (95%CI)	Psych. Therapy (visits) Mean (95%CI)	Medication (people) Mean (95%CI)
47%	381.72 (380.51 , 382.93)	1031.11 (1026.02 , 1036.20)	180.09 (179.28 , 180.97)	58.45 (58.16 , 58.74)
65%	381.60 (380.44 , 382.75)	1746.90 (1740.51 , 1753.29)	301.80 (300.73 , 302.87)	99.21 (98.84 , 99.58)
80%	383.35 (382.22 , 384.48)	2105.07 (2097.72 , 2112.41)	363.55 (362.32 , 364.77)	119.58 (119.16 , 120.00)
**Service Coverage**	**Psychiatrist (visits) Mean (95%CI)**	**CMHTeam (visits) Mean (95%CI)**	**CRHTT (people) Mean (95%CI)**	**Inpatient (weeks) Mean (95%CI)**
47%	126.97 (126.02 , 127.91)	387.06 (384.27 , 389.85)	82.63 (82.18 , 83.09)	75.90 (76.42 , 77.38)
65%	216.23 (215.06 , 217.40)	615.66 (612.12 , 619.20)	92.94 (92.44 , 93.43)	58.61 (58.15 , 59.08)
80%	260.04 (258.62 , 261.45)	722.81 (718.60 , 727.02)	93.54 (92.99 , 94.10)	43.05 (42.58 , 43.51)

Table S2. Estimation for service costs for depression

Service Coverage (%)	Description	N	GP	Medication	Psychological Therapy	Psychiatrist	Inpatient	CMHTeam	Grand Total (£)
47%	Total prevalence	382							
	Cost (Average £)		38.00	73.84	55.00	108.00	407.00	197.00	
	Service use		1,031	58	180	127	531	387	
	Costs(£; pop size 5000)		39,182.18	4,315.95	9,904.95	13,712.76	216,239.10	76,250.82	359,605.76
	Costs(£; ABUHB adults 476,139)		3,731,319.00	411,007.73	943,248.39	1,305,866.13	**20,592,449.49**	7,261,365.59	**34,245,256.33**
65%	Total prevalence	382							
	Cost (Average in £)		38.00	73.84	55.00	108.00	407.00	197.00	
	Service use		1,747	99	302	216	410	616	
	Costs(in £; pop size 5000)		66,382.20	7,325.67	16,599.00	23,352.84	166,979.89	121,285.02	401,924.62
	Costs(in £; ABUHB adults 476,139)		6,321,576.91	697,623.21	1,580,722.77	2,223,890.95	**15,901,494.92**	11,549,972.45	**38,275,281.22**
80%	Total prevalence	383							
	Cost (Average in £)		38.00	73.84	55.00	108.00	407.00	197.00	
	Service use		2,105	120	364	260	301	723	
	Costs(in £; pop size 5000)		79,992.66	8,829.79	19,995.25	28,084.32	122,649.45	142,393.57	401,945.04
	Costs(in £; ABUHB adults 476,139)		7,617,701.01	840,860.64	1,904,147.66	2,674,469.79	**11,679,907.12**	13,560,139.67	**38,277,225.89**

The medicine cost was derived from [1], other costs were derived from [2].

The results are averaged for yearly costs.

The CRHTT was not included in the evaluation due to difficulty of getting the reference for the yearly costs of individuals having contact with the CRHTT.

Table S3: Variable description for mild depression SD model

Type of Variables	Description	Equation
Stocks		
Initial GP visits (X_1)	Stock representing the number of individuals having consultation with GP at the initial stage.	$\frac{\partial X_1}{\partial t} = I_1 - O_1$
Need further treatment (X_2)	Transition stock for those who need further treatment after the initial GP visits.	$\frac{\partial X_2}{\partial t} = I_2 - O_2$
Total used medication (X_3)	To count the total number of patients who used medication.	$\frac{\partial X_3}{\partial t} = I_3$
Total GP visits (X_4)	To count the total number of GP visits generated by the patients.	$\frac{\partial X_4}{\partial t} = I_4$
Total Psychological Therapy visits (X_5)	To count the total number of Psychological Therapy visits generated by the patients.	$\frac{\partial X_5}{\partial t} = I_5$
Flows		
Inflow to initial GP visits (I_1)	The rate of individuals who enter the GP consultations.	$I_1 = a_1$
Outflow from Initial GP visits (O_1)	The rate of individuals who finish with their GP consultation.	$O_1 = X_1/T_1$
Inflow to further treatment (I_2)	The rate of individuals who need further treatment after their first GP consultation.	$I_2 = O_1 * p_2$
Outflow from Need further treatment (O_2)	The rate of individuals who will go to have further treatment.	$O_2 = X_2/T_2$
Inflow to total used medication (I_3)	The rate of individuals who used medication.	$I_3 = O_2 * p_4$
Inflow to Total GP visits (I_4)	The rate represents the number of GP visits needed by the individuals. The rate from a_5 represents the end of treatment GP visits.	$I_4 = a_3 + a_4 + a_5$
Inflow to Psychological Therapy visits (I_5)	The rate representing the number of therapy visits needed.	$I_5 = a_5 * p_5$
Auxiliary variables		
Initial demand (a_0)	Exogenous variable generated from the Agent Based model to update the number of individuals affected by mild depression every time step.	
Enter treatment (a_1)	Endogenous variable representing the rate of individuals entering the health service.	$a_1 = a_0 * p_0$

Table S3 continued

Type of Variables	Description	Equation
Not treated (a_2)	Endogenous variable representing the rate of individuals not entering the health service.	$a_2 = a_0 - a_1$
Further GP visits (a_3)	Endogenous variable to account for the rate of additional GP visits due to the use of medication.	$a_3 = I_3 * p_3$
Enter initial GP visits (a_4)	Endogenous variable to account for the total number of GP visits at the initial stage.	$a_4 = a_1 * p_1$
Need Psychological Therapy (a_5)	Endogenous variable to account for the rate of individuals with depression who need psychological therapy.	$a_5 = O_2 * p_6$

Table S4: Parameter description for mild depression SD model

Parameter	Description
Time in initial GP (T_1)	Time spent for the initial consultation.
Observation time (T_2)	Time representing observation duration if the affected individuals responded to the initial GP consultation.
Proportion enter service (p_0)	A constant representing the proportion of individuals who enter the service.
Number initial GP visits (p_1)	a constant representing the number of GP visits needed at the initial stage.
Fraction need further treatment (p_2)	A constant representing the proportion of individuals who need further treatment after the initial GP consultation.
Number further GP visits (p_3)	A constant representing the number of GP visits needed as a result of further treatment.
Fraction need medication (p_4)	A constant representing the fraction of individuals who need medication.
Number Psychological Therapy visits (p_5)	A constant representing the number of visits needed to have Psychological Therapy.
Fraction need Psychological Therapy (p_6)	A constant representing the proportion of individuals who need Psychological Therapy.

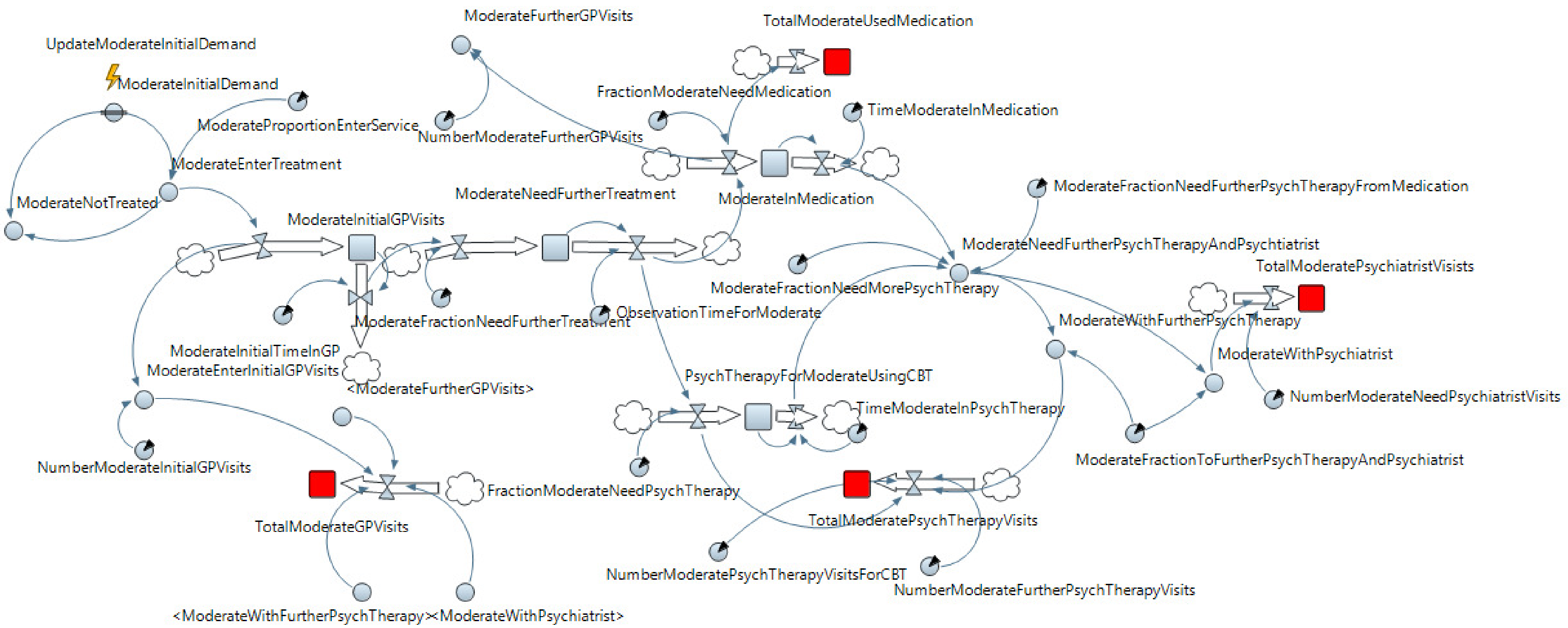

Figure S1. SD Model for Moderate Depression, developed in AnyLogic

Table S5: Variable description for moderate depression SD model

Type of Variables	Description	Equation
Stocks		
Initial GP visits (X_1)	Stock representing the number of individuals having consultation with GP at the initial stage.	$\frac{\partial X_1}{\partial t} = I_1 - O_1$
Need further treatment (X_2)	Transition stock for those who need further treatment after the initial GP visits.	$\frac{\partial X_2}{\partial t} = I_2 - O_2$
In medication (X_3)	Stock representing the number of patients who used medication.	$\frac{\partial X_3}{\partial t} = I_3 - O_3$
Psychological Therapy after CBT (X_4)	Stock representing the number of patients who have psychological therapy after having CBT.	$\frac{\partial X_4}{\partial t} = I_4 - O_4$
Total used medication (X_5)	To count the total number of patients who used medication.	$\frac{\partial X_5}{\partial t} = I_5$
Total Psychological Therapy visits (X_6)	To count the total number of Psychological Therapy visits generated by the patients.	$\frac{\partial X_6}{\partial t} = I_6$
Total Psychiatrist visits (X_7)	To count the total number of Psychiatrist visits generated by the patients.	$\frac{\partial X_7}{\partial t} = I_7$
Total GP visits (X_8)	To count the total number of GP visits generated by the patients.	$\frac{\partial X_8}{\partial t} = I_8$
Flows		
Inflow to initial GP visits (I_1)	The rate of individuals who enter the GP consultations.	$I_1 = a_1$
Outflow from Initial GP visits (O_1)	The rate of individuals who finish with their GP consultation.	$O_1 = X_1/T_1$
Inflow to further treatment (I_2)	The rate of individuals need further treatment after their first GP consultation.	$I_2 = O_1 * p_2$
Outflow from Need further treatment (O_2)	The rate of individuals who will go to have further treatment.	$O_2 = X_2/T_2$
Inflow in medication (I_3)	The rate of individuals who use medication.	$I_3 = O_2 * p_3$
Outflow from using medication (O_3)	The rate of individuals who finish using medication.	$O_3 = X_3/T_3$
Inflow to Psychological Therapy (I_4)	The rate of individuals who need Psychological Therapy with using CBT.	$I_4 = O_2 * p_6$
Outflow from Psychological Therapy with CBT (O_4)	The rate of individuals who finish Psychological Therapy with CBT.	$O_4 = X_4/T_4$
Inflow to total use medication (I_5)	The rate to count total individuals who used medication	$I_5 = I_3$

Table S5 continued

Type of Variables	Description	Equation
Inflow to total psychological Therapy visits (I_6)	The rate to count the total number of psychological Therapy visits	$I_6 = (I_4 * p_7) + (a_6 * p_8)$
Inflow to total Psychologist visits (I_7)	The rate to count the total number of Psychologist visits	$I_7 = a_7 * p_9$
Inflow to total GP visits (I_8)	The rate to count the total number of GP visits	$I_8 = a_3 + a_4 + a_6 + a_7$
Auxiliary variables		
Initial demand (a_0)	Exogenous variable generated from the Agent Based model to update the number of individuals affected by moderate depression every time step.	
Enter treatment (a_1)	Endogenous variable representing the rate of individuals entering the health service.	$a_1 = a_0 * p_0$
Not treated (a_2)	Endogenous variable representing the rate of individuals not entering the health service.	$a_2 = a_0 - a_1$
Enter initial GP visits (a_3)	Endogenous variable to account for the total number of GP visits at the initial stage.	$a_3 = I_1 * p_1$
Further GP visits (a_4)	Endogenous variable to account for additional number of GP visits due to further treatment	$a_4 = I_3 * p_4$
Need further Psychological Therapy and Psychiatrist (a_5)	Endogenous variable to count the number of individuals who need further treatment from Psychological Therapy and Psychiatrist	$a_5 = (O_3 * p_{11}) + (O_4 * p_5)$
Further Psychological Therapy (a_6)	Endogenous variable to count the number of individuals who need additional Psychological Therapy	$a_6 = a_5 * p_{10}$
With Psychiatrist (a_7)	Endogenous variable to count the number of individuals who need Psychiatrist	$a_7 = a_5 * p_{10}$

Table S6: Parameter description for moderate depression SD model

Parameter	Description
Time in initial GP (T_1)	Time spent for the initial consultation.
Observation time (T_2)	Time representing observation duration if the affected individuals responded to the initial GP consultation.
Time in medication (T_3)	Time spent in medication treatment.
Time in Psychological Therapy (T_4)	Time spent for having Psychological Therapy.
Proportion enter service (p_0)	A constant representing the proportion of individuals who enter the service.
Number initial GP visits (p_1)	A constant representing the number of GP visits needed at the initial stage.
Fraction need further treatment (p_2)	A constant representing the proportion of individuals who need further treatment after initial GP consultation.
Fraction need medication (p_3)	A constant representing the fraction of individuals who need medication.
Number further GP visits (p_4)	A constant representing the number of GP visits needed as a result from further treatment.
Fraction need more Psychological Therapy (p_5)	A constant representing the proportion of individuals who need additional Psychological Therapy
Fraction need Psychological Therapy (p_6)	A constant representing the proportion of individuals who need Psychological Therapy.
Number Psychological Therapy visits for CBT (p_7)	A constant representing the number of visits needed for having Psychological Therapy.
Number further Psychological Therapy visits (p_8)	A constant representing the number of visits needed for having additional Psychological Therapy.
Number need Psychiatrist visits (p_9)	A constant representing the number of Psychiatrist visits needed.
Fraction to further Psychiatrist and Psychological Therapy visits (p_{10})	A constant representing the proportion of individuals who need additional visits to Psychiatrist and Psychological Therapy.
Fraction need further Psychological Therapy from medication (p_{11})	A constant representing the proportion of individuals who need additional Psychological Therapy from having medication.

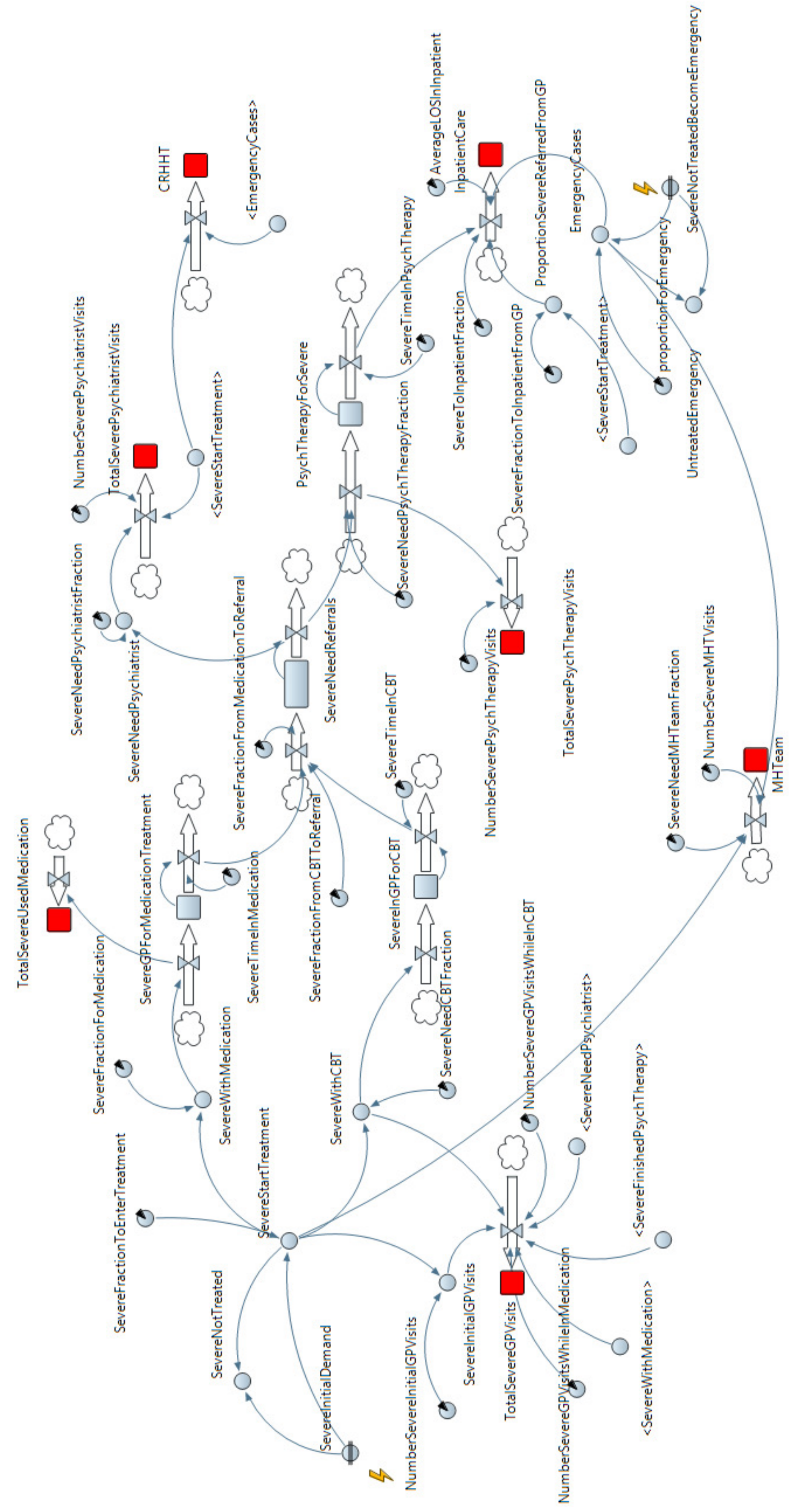

Figure S2. SD Model for Severe Depression, developed in AnyLogic

Table S7: Variable description for severe depression SD model

Type of Variables	Description	Equation
Stocks		
GP for CBT (X_1)	Stock representing the number of individuals having consultation with GP for having CBT.	$\frac{\partial X_1}{\partial t} = I_1 - O_1$
GP for medication treatment (X_2)	Stock representing the number of individuals having consultation with GP for having medication.	$\frac{\partial X_2}{\partial t} = I_2 - O_2$
Need referrals (X_3)	Transition stock representing the number of patients who need referrals for further treatment.	$\frac{\partial X_3}{\partial t} = I_3 - O_3$
Psychological Therapy (X_4)	Stock representing the number of patients having Psychological Therapy.	$\frac{\partial X_4}{\partial t} = I_4 - O_4$
Total GP visits (X_5)	To count the total number of GP visits.	$\frac{\partial X_5}{\partial t} = I_5$
Total used medication (X_6)	To count the total number of individuals having medication.	$\frac{\partial X_6}{\partial t} = I_6$
Total Psychological Therapy visits (X_7)	To count the total number of Psychological Therapy visits generated by the patients.	$\frac{\partial X_7}{\partial t} = I_7$
Total Psychiatrist visits (X_8)	To count the total number of Psychiatrist visits generated by the patients.	$\frac{\partial X_8}{\partial t} = I_8$
Inpatient care (X_9)	To count the total number of inpatient care in weeks.	$\frac{\partial X_8}{\partial t} = I_9$
CRHTT (X_{10})	To count the total number of patients served by the Crisis Resolution Home Treatment Team.	$\frac{\partial X_{10}}{\partial t} = I_{10}$
MHTeam (X_{11})	To count the total number of MHTeam visits.	$\frac{\partial X_{11}}{\partial t} = I_{11}$
Flows		
Inflow to GP for CBT (I_1)	The rate of individuals who enter the GP consultations for having CBT.	$I_1 = a_3$
Outflow from GP for CBT (O_1)	The rate of individuals who finish with GP consultation for having CBT.	$O_1 = X_1/T_1$
Inflow to GP for medication treatment (I_2)	The rate to count individuals who need GP visits for having medication.	$I_2 = a_4$
Outflow from GP for medication treatment (O_2)	The rate of individuals finishing GP visits due to having medication.	$O_2 = X_2/T_2$

Table S7 continued

Type of Variables	Description	Equation
Inflow to referrals (I_3)	The rate of individuals who need referrals for more treatment.	$I_3 = (O_1 * p_6) + (O_2 * p_7)$
Outflow from referrals(O_3)	The rate of individuals referred to further treatment.	$O_3 = X_3$
Inflow to Psychological Therapy (I_4)	The rate of individuals who need Psychological Therapy.	$I_4 = O_3 * p_8$
Outflow from Psychological Therapy (O_4)	The rate of individuals who finished Psychological Therapy.	$O_4 = X_4/T_3$
Inflow to total GP visits (I_5)	The rate to count total number of GP visits	$I_5 = a_5 + (a_3 * p_5) + (a_3 * p_5) + a_{10} + a_4 + O_4$
Inflow to total used medication (I_6)	The rate to count total number of individuals who used medication	$I_6 = I_2$
Inflow to total Psychological Therapy visits (I_7)	The rate to count the total number of Psychological Therapy visits	$I_7 = I_4 * p_9$
Inflow to total Psychiatrist visits (I_8)	The rate to count the total number of Psychiatrist visits	$I_8 = a_{10} * p_{11}$
Inflow to total inpatient care (I_9)	The rate to count the total number of inpatient use in weeks	$I_9 = ((O_4 * p_{12}) + a_9 + a_7) * T_4$
Inflow to CRHTT (I_{10})	The rate to count the total number of individuals who have in contact with CRHTT	$I_{10} = a_1 + a_7$
Inflow to total MHteam visits (I_{11})	The rate to count the total number of MH team visits	$I_{11} = a_1 * p_{10} * p_{16} * p_{15}$
Auxiliary variables		
Initial demand (a_0)	Exogenous variable generated from the Agent Based model to update the number of individuals affected by severe depression every time step.	
Enter treatment (a_1)	Endogenous variable representing the rate of individuals entering the health service.	$a_1 = a_0 * p_0$
Not treated (a_2)	Endogenous variable representing the rate of individuals not entering the health service.	$a_2 = a_0 - a_1$
With CBT (a_3)	Endogenous variable to account for the number of individuals having CBT.	$a_3 = a_1 * p_1$
With medication (a_4)	Endogenous variable to account for the number of individuals having medication.	$a_4 = a_1 * p_2$

Table S7 continued

Type of Variables	Description	Equation
Number initial GP visits (a_5)	Endogenous variable to count the number of individuals having initial consultation with GP.	$a_5 = a_1 * p_3$
Not treated become emergency (a_6)	Exogenous variable generated from the Agent Based model to count for the number of individuals who need emergency treatment.	
Emergency cases (a_7)	Endogenous variable to count the number of individuals who need emergency service	$a_7 = a_6 * p_{14}$
Untreated emergency (a_8)	Endogenous variable to count the number of individuals who cannot be treated in emergency service .	$a_8 = a_6 - a_7$
proportion referred from GP (a_9)	Endogenous variable to count the number of individuals who need emergency service referred from GP.	$a_9 = a_1 * p_{13}$
Need psychiatrist (a_{10})	Endogenous variable to count the number of individuals who need Psychiatrist	$a_{10} = O_3 * p_{10}$

Table S8: Parameter description for severe depression SD model

Parameter	
Time in CBT (T_1)	Time spent for having CBT.
Time in medication (T_2)	Time spent when using medication.
Time in Psychological Therapy (T_3)	Time spent having Psychological Therapy.
Time in inpatient stay (T_4)	Average time spent in the inpatient service.
Fraction to enter treatment (p_0)	A constant representing the proportion of individuals who enter the service.
Need CBT fraction (p_1)	A constant representing the proportion of individuals who need CBT treatment.
Fraction for medication (p_2)	A constant representing the proportion of individuals who use medication.
Number initial GP visits (p_3)	A constant representing the number of GP visits at initial stage.
Number GP visits while in medication (p_4)	A constant representing the number of GP visits needed as a result of using medication.
Number GP visits while in CBT (p_5)	A constant representing the number of GP visits needed while having CBT.
Fraction from CBT to referral (p_6)	A constant representing the proportion of individuals who need referral after having CBT.
Fraction from medication to referral (p_7)	A constant representing the proportion of individuals who need referral after having medication.
Need Psychological Therapy fraction (p_8)	A constant representing the proportion of individuals who need Psychological Therapy.
Number Psychological Therapy visits (p_9)	A constant representing the number of Psychological Therapy visits needed.
Need Psychiatrist fraction (p_{10})	A constant representing the proportion of individuals who need Psychiatrist.
Number Psychiatrist visits (p_{11})	A constant representing the number of visits needed for Psychiatrist.
To inpatient fraction (p_{12})	A constant representing the proportion of individuals who need inpatient care.
Fraction to inpatient from GP (p_{13})	A constant representing the proportion of individuals who need inpatient care directly from GP.

Table S8 continued

Parameter	Description
Proportion for emergency (p_{14})	A constant representing the proportion of individuals having emergency service.
Need MH Team fraction (p_{15})	A constant representing the proportion of individuals who need Mental Health Team service.
Number MH visits (p_{16})	A constant representing the number of visits when receiving service from the MH Team.

Table S9: List of parameters for System Dynamics model

**Description**	**est. value**	**Source**
**SD Model (Mild)**		
Time in initial GP	4 weeks	[1]
Observation time	2 weeks	[1]
Proportion enter service	varies	scenario for treatment coverage
Number initial GP visits	4 visits	[1]
Fraction need further treatment	30%	[1]
Number further GP	6 visits	[1]
Fraction need medication	0.67 (20% of 30%)	[1]
Number Psychological Therapy visits	6 visits	[1]
Fraction need Psychological Therapy	0.33 (10% of 30%)	[1]
**SD Model (Moderate)**		
Time in initial GP	4 weeks	[1]
Observation time	1 week	adapted from [1]
Time in medication	46 weeks	[1]
Time in Psychological Therapy	6 weeks	[1]
Proportion enter service	varies	scenario for treatment coverage
Number initial GP visits	4 visits	[1]
Fraction need further treatment	70%	[1]
Fraction need medication	0.57 (40% of 70%)	[1]
Number further GP visits	8 visits	[1]

Table S9 continue

Description	est. value	Source
Fraction need more Psychological Therapy	0.33 (10% of 30%)	[1]
Fraction need Psychological Therapy	0.43 (30% of 70%)	[1]
Number Psychological Therapy visits for CBT	6 visits	[1]
Number Further Psychological Therapy visits	6 visits	[1]
Number need Psychiatrist visits	6 visits	[1]
Fraction on further Psychiatrist and Psychological Therapy visits	0.5	adapted from [1]
Fraction need further Psychological Therapy from medication	0.25 (10% of 40%)	[1]
**SD Model (Severe)**		
Time in CBT	16 weeks	[1, 2]
Time in medication	56 weeks	[1, 2]
Time in Psychological Therapy	16 weeks	[2]
Fraction to enter treatment	varies	[1]
Need CBT fraction	10%	[1]
Fraction for medication	90%	[1]
Number initial GP visits	4 visits	[1]
Number GP visits while in medication	12 visits	[1]
Number GP visits while in CBT	6 visits	[1]
Fraction from CBT to referrals	70%	[1]
Fraction from medication to referrals	70%	[1]
Fraction need Psychological Therapy	0.43 (30% of 70%)	[1]
Number Psychological Therapy visits	10 visits	[1]
Need Psychiatrist fraction	0.57 (40% of 70%)	[1]
Number Psychiatrist visits	10 visits	[1]
To inpatient fraction	0.17 (5% of 30%)	[1]
Fraction to inpatient from GP	0.05	[1]

Table S9 continue

Description	est. value	Source
Proportion for emergency	0.5	scenario for treatment coverage
Need MH Team fraction	1	adapted from [1]
Number MT Team visits	10 visits	[1]
Time in inpatient stay	10.25 days (1.5 weeks)	[3]

[1] [3],

[2] [4],

[3] The average mean value from Anuerin Bevan University Health Board.

REFERENCES

[1]King's Fund. Paying the price: The cost of mental health in England to 2026 (2008). Accessed 29/10/2018.

[2]Curtis LA, Burns A. Unit Costs of Health and Social Care 2017 (2017). Personal Social Services Research Unit, University of Kent, Canterbury, 260 pp. ISBN 978-1-911353-04-1, Accessed 29/10/2018.

[3]Andrews, G and the TOLKIEN II Team. *Tolkien II: A needs-based, costed stepped-care model for mental health services* (Sydney: WHO Collaborating Centre for Classification in Mental Health) (2006).

[4]National Collaborating Centre for Mental Health. *Depression: the NICE guideline on the treatment and management of depression in adults (updated edition)* (London: The British Psychological Society & The Royal College of Psychiatrists) (2010).
